# Role of Complementary and Alternative Medicine in Cardiovascular Diseases

**DOI:** 10.1155/2013/142898

**Published:** 2013-06-17

**Authors:** Waris Qidwai, Peng Nam Yeoh, Victor Inem, Kashmira Nanji, Tabinda Ashfaq

**Affiliations:** ^1^Family Medicine Department, Aga Khan University, Karachi, Pakistan; ^2^International Medical University, Kuala Lumpur, Malaysia; ^3^Department of Family Medicine, Lagos University, Nigeria

*Introduction.* This special issue focuses on the role of complementary and alternative medicine (CAM) in cardiovascular diseases (CVD). The role of CAM in healthcare specifically in cardiovascular diseases (CVDs) has been a contentious issue for centuries. With demographic shifts, urbanization, and changing lifestyles, disease burden of cardiovascular diseases (CVDs) has increased dramatically and can further increase in the future. Despite the growing popularity of CAM therapies, limited information is available regarding patterns of use of CAM therapies in cardiovascular diseases.

The definition of CAM has continued to evolve. As defined by the National Center of complementary and alternative medicine (NCCAM), “it is a group of diverse medical and healthcare systems, practices, and products that are not generally considered part of conventional medicine.” The 5 domains of CAM as classified by the NCCAM are whole medical systems (e.g., homeopathy, and ayurvedic medicine), mind-body interventions (e.g., yoga, meditation, and hypnotherapy), biologically based therapies (e.g., herbal treatments, mega-dose vitamins), manipulative and body-based methods (e.g., chiropractic therapy), and energy therapies (e.g., Reiki, and magnetic therapy).

Over the last several decades, the use of CAM has become increasingly popular in both developed and the developing countries. A high proportion of patients using CAM believe CAM has remedial benefits and are safe compared to their prescribed treatments; this serves as a strong motivational factor for both present and future use of CAM. In addition, patients with CVD might be more likely to seek CAM treatments to decrease the psychological stress associated with this condition. Misconceptions regarding their efficacy have largely driven the popularity of these products whereas the adverse effects have been underreported. In disadvantaged societies where access to biomedical services is poor, the reliance on traditional/herbal medicines is more. In affluent population CAM is more used for disease prevention and health promotion. Data from the National Health Interview Surveys (NHIS) reported that 38% of adults in the USA were using CAM therapy in 2007 and among those 36% had CVD. 

CVD patients are often unwilling to inform their medical practitioners of CAM use and the majority of attending physicians do not discuss CAM use with their patients. Since many commonly used CAM products have the potential to interfere with the intended action of concomitant prescription medications, this could lead to serious drug interactions. In addition, the use of CAM may have negative impact on the compliance with prescription medications. 

A number of CAM therapies have purported cardiovascular effects; but most research on these products is either inconclusive, conflicting, or shows no benefit for their use. Several systematic reviews and meta-analysis on the effectiveness and possible side effects of CAM interventions suggest that some approaches may be beneficial as adjuncts to conventional management of cardiovascular disease, but no evidence exists to support their role as primary treatment. 

Dietary supplements (fish oil, coenzyme Q10, garlic, etc.) are among the most commonly used treatment modalities in patients with CVD. Fish oil supplements are accepted as a part of the treatment regimen for elevated serum triglycerides and the maintenance of vascular wall health. However, the efficacy of vitamin E has been questionable.

Another intervention of CAM, that is, mind-body therapies (relaxation, stress management, meditation, etc.) have minimal side effects. However, in some countries unavailability of trained professionals in the field poses hindrance in its usage. Several styles of meditation have been tested and found to reduce blood pressure, improve heart rate, and even provide survival benefit. Evidence-based trials have been supportive of the conclusion that yoga can lower blood pressure and improve physical fitness. 

This one issue cannot answer all the questions regarding the safety, quality, and effectiveness of CAM therapies in CVDs. However, the main purpose of this issue is to open the communication line between patients and physicians on CAM use. It also illustrates the necessity of more rigorous researches to determine the precise pharmacological effects and long-term benefits on cardiovascular morbidity and mortality with CAM usage. 

Altogether, 27 papers were submitted for publication, out of which 19 papers were accepted. The articles in this issue represent a wide range of therapeutic approaches of CAM in preventing cardiovascular diseases. There are papers on extracts of herbal plants such as *Nigella sativa*, extract of black chokeberry, *Salvia miltiorrhiza*, polyphenol and *Pueraria lobata*, and their cardioprotective role in treating hypertension. Ethanolic extract of black chokeberry fruits has a potential role as prophylactic agent but can also function as a nutritional supplement in the management of arterial hypertension. In addition, a study on the use of repeatedly heated oil (a common practice in Asian countries) concluded that it has the predisposing factor of atherosclerosis leading to cardiovascular diseases. Therefore, it is advisable to avoid the consumption of repeatedly heated palm oil. 

This special issue also has a number of reviews on the role of CAM in preventing CVD. There is a review on the role of garlic in cardiovascular diseases: treatment and prevention which concluded that garlic can be used as an adjuvant with lipid lowering drugs for control of lipids. Moreover, another evidence-based review discusses CAM and CVDs; this review recommends that more rigorous researches are needed to determine the precise physiologic effects and long-term benefits on cardiovascular morbidity and mortality with CAM usage. In addition, there is a review on Chinese herbal medications (CHM) for hypertension. This review on 10 systematic reviews found that the majority of the RCTs (randomised controlled trials) do not include primary endpoints and therefore their conclusions remain uncertain. Another review on a traditional Chinese herbal formula, Zhen Gan Xi Feng Decoction, appears to be effective in improving blood pressure and symptoms in patients with essential hypertension.

The edition also includes a paper on protein kinase II signal transduction pathway that inhibits cardiac arrhythmias in rats with myocardial infarction. Another study on palm tocotrienol-rich fraction found that it was comparable to folate in reducing high-methionine diet-induced plasma hyperhomocysteinemia, aortic oxidative stress, and inflammatory changes in rats. 

*Conclusion*. The articles presented in this issue represent the recognition of CAM's role in CVD patients. Nevertheless, better education of patients and medical practitioners is needed to improve the understanding of the risks and benefits of CAM use in CVD patients. Further pieces of evidence are required to determine the impacts of CAM use in CVD patients, particularly its clinical and prognostic impact when used in conjunction with prescription medicines. An open dialogue between healthcare professionals and patients regarding intended or present CAM use is also warranted.

*Acknowledgments*. We hope that this special issue informs and stimulates thinking about the rationale use of CAM in CVD patients. We also hope that readers will find the papers included in this issue a valuable contribution to the field and it reflects the recent trends. We would like to thank the contributors to this special issue for their insightful papers. We would also like to acknowledge the many reviewers for their detailed comments and constructive suggestions. I wish to express my gratitude to all the Guest Editors for encouraging this project throughout, and meticulously carrying out the numerous and often arduous tasks involved with this project.

*Waris Qidwai* *Waris Qidwai* *Peng Nam Yeoh* *Peng Nam Yeoh* *Victor Inem* *Victor Inem* *Kashmira Nanji* *Kashmira Nanji* *Tabinda Ashfaq* *Tabinda Ashfaq*

